# Is there a role for the platelet-to-lymphocyte ratio in chronic lymphocytic leukemia?

**DOI:** 10.4155/fsoa-2018-0061

**Published:** 2018-10-05

**Authors:** Ziad Bakouny, Elie El Rassy, Fares Yared, Antoine Abi Lutfallah, Marwan Ghosn, Fadi Farhat, Joseph Kattan

**Affiliations:** 1 Department of Hematology–Oncology, Hotel-Dieu de France University Hospital, Faculty of Medicine, Saint Joseph University, Beirut, Lebanon

**Keywords:** chronic lymphocytic leukemia, CLL, platelet-to-lymphocyte ratio, PLR

## Abstract

**Aim:**

The rationale for platelet-to-lymphocyte ratio (PLR) in chronic lymphocytic leukemia (CLL) is that both the platelet and lymphocyte counts are affected by the CLL pathogenesis and could influence treatment decision-making.

**Methods:**

Demographic and clinical data of CLL patients diagnosed at our institution between 1989 and 2013 were collected. Cox regression models were used to evaluate the role of PLR in the duration of watchful waiting, postdiagnosis survival and postchemotherapy survival.

**Results:**

The data of 100 patients with CLL were reviewed for this study. The PLR correlated only to watchful waiting in the univariable analysis (Hazard ratio = 0.48 [0.32–0.73]; p = 0.018). In the multivariable analysis, the duration of watchful waiting was determined by Binet staging and lymphocyte count (p < 0.001). The postdiagnosis survival was determined by age (p = 0.002) and lymphocyte count (p = 0.010).

**Conclusion:**

The PLR did not seem to act as a prognostic biomarker for CLL.

## Lay abstract

Patients with chronic lymphocytic leukemia (CLL), an incurable but often indolent hematological cancer, often have increased lymphocytes and decreased platelets. We investigated whether the ratio of platelet-to-lymphocyte count can be used as a biomarker to predict the prognosis of patients with CLL. An increase in the platelet-to-lymphocyte ratio was found to be related to a decrease in the duration of watchful waiting (the duration between the diagnosis of CLL and the administration of chemotherapy to the patient). However, the ratio was not found to be related to patient survival and therefore, based on this study, it cannot be considered as a biomarker for the prognosis of CLL.

Chronic lymphocytic leukemia (CLL) is an incurable neoplastic disorder that is characterized by a gradual accumulation of small and mature B cells [1]. These cells are replicationally dormant yet they accumulate in the bone marrow and the peripheral blood, mainly due to extrinsic survival signals emitted from the microenvironment [2]. In comparison with normal B cells, the leukemic cells are selectively rescued from apoptosis by inflammatory signals [3]. Indeed, the role of inflammation clearly manifests in CLL patients, who seem to present a wide range of manifestations, that are typically encountered in chronic inflammatory diseases [4]. Multiple studies have shown that the platelet-to-lymphocyte ratio (PLR), an inflammatory biomarker, is a prognostic factor for solid tumors [5]. Moreover, PLR finds its rationale in CLL where both the platelet and lymphocyte counts are directly correlated to CLL pathogenesis and influence patient management [6]. Therefore, we developed PLR as a prognostic parameter in CLL and investigated its prognostic value across different outcomes.

## Materials & methods

This is an institutional review board approved retrospective study to evaluate the role of PLR as a biomarker in CLL. This study included all treatment-naive patients diagnosed with CLL at three clinics affiliated to Hotel Dieu de France University Hospital between January 1989 and 2013. Patients with auto-immune hematologic manifestations were not included in this analysis. Each record was confirmed for the diagnosis of CLL according to the following criteria: presence of lymphocytosis above 5 × 10^9^/l with at least 50% B-lymphocytes and a flow cytometry signature characterized by CD5^+^, CD19^+^, CD23^+^, CD20 (dim) and surface immunoglobulin (dim). The small lymphocytic lymphoma patients did not require a lymphocytosis above 5 × 10^9^/l with at least 50% B-lymphocytes [6]. The records were also reviewed for epidemiologic data and clinical presentation. The laboratory tests at diagnosis were collected, before any treatment, including the lymphocyte count (number/mm^3^) and platelet count (number/mm^3^). We calculated the PLR by dividing the absolute count of platelets to that of lymphocytes at diagnosis (when both expressed in number/mm^3^). All patients were classified according to the Binet system at diagnosis. For all patients, three survival end points were defined. First, duration of watchful waiting was defined as the time from diagnosis until the start of chemotherapy or death. Second, postdiagnosis survival (or overall survival [OS]) was defined as the duration from diagnosis until death. Third, postchemotherapy survival was defined as the duration from the start of chemotherapy until death. All three end points were reported in number of days. Patients who were lost to follow-up or who did not suffer any of the events for a given end point at last follow-up were censored for that end point.

Continuous variables were summarized by their medians and ranges. Categorical variables were summarized by number and percentage of patients within each category. SPSS Statistics version 20.0 (IBM Corporation, NY, USA) and XlStat version 2017.1 (Addinsoft, Paris, France) were used for statistical analysis. All continuous variables were analyzed as such and not categorized. Univariable Cox regression models were computed while considering duration of watchful waiting, postdiagnosis survival and postchemotherapy survival as dependent variables, and demographic, laboratory and disease characteristics as independent variables. Multivariable stepwise Cox regression models were subsequently computed with all independent variables included. Effect sizes were reported as hazard ratios (HR) for univariable tests and adjusted hazard ratios (HRa) for multivariable tests, with their 95% CIs. All tests were two-tailed and considered statistically significant for p < 0.05.

## Results

A total of 100 CLL patients with a median age of 64 years (range 36–89) were included in this study and their characteristics are summarized in Table 1. At diagnosis, the median absolute count of lymphocytes was 18,500/mm^3^ (range 1596–290,000/mm^3^) and platelets 199,500/mm^3^ (range 14,000–458,000/mm^3^). The median platelet-to-lymphocyte ratio was 11.27 (range 0.14–159.77). The Binet stage was A in 64.1%, B in 17.4% and C in 18.5%. The most common indications for treatment included anemia/thrombocytopenia (33%) and bulky lymph nodes (18%). During follow-up, 41% of this sample required treatment. All patients received a rituximab-based regimen combining bendamustin with rituximab in the frail patients and fludarabine–cyclophosphamide with rituximab in the fit patients.

**Table 1.** **Demographic characteristics of the included patients.**

**Patients’ characteristics**	**Percentage (median)**

Age in years – median (range)	64 (36–89)

Gender – n (%)	

Male	60 (60%)

Female	40 (40%)

Binet stage – n (%)	

A	59 (64.1%)

B	16 (17.4%)

C	17 (18.5%)

Missing	8

Splenomegaly – n (%)	

No	71 (72.4%)

Yes	27 (27.6%)

Missing	2

Hepatomegaly – n (%)	

No	91 (92.9%)

Yes	7 (7.1%)

Missing	2

Lymphocyte count/mm^3^ – median (range)	18,500 (1956–290,000)

Platelet count/mm^3^ – median (range)	199,500 (14,000–458,000)

Platelet to lymphocyte ratio – median (range)	11.27 (0.14–159.77)

**Figure 1.**
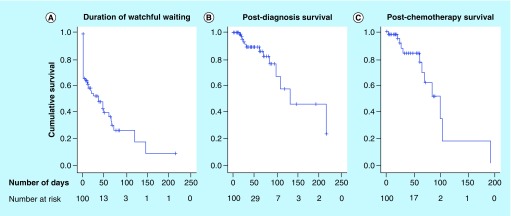
**Kaplan–Meier survival curves for the patients with chronic lymphocytic leukemia included in this study.** Kaplan–Meier survival curves for the patients with chronic lymphocytic leukemia included in this study for **(A)** duration of watchful waiting, **(B)** post-diagnosis survival, and **(C)** post-chemotherapy survival.

The Kaplan–Meier curves of watchful waiting, postdiagnosis survival and postchemotherapy survival are represented in Figure 1. The results of the univariable and multivariable Cox regression analyses are presented in Table 2. In the univariable analysis, patients with Binet stages B (HR = 3.66 [1.77–7.56]; p < 0.001) or C (HR = 6.46 [3.02–13.82]; p < 0.001) had significantly shorter durations of watchful waiting compared with subjects with Binet stage A. For each increase of the lymphocyte count by 1000/mm^3^, patients had significantly decreased duration of watchful waiting (HR = 1.008 [1.004–1.012]; p < 0.001). For each increase of the platelet count by 100,000/mm^3^, patients had significantly increased duration of watchful waiting (HR = 0.48 [0.32–0.73]; p = 0.001). For each increase of the PLR by one unit, the duration of watchful waiting increased significantly (HR = 0.48 [0.32–0.73]; p = 0.018) but PLR did not correlate with the two other survival outcomes.

**Table 2.** **Univariable and multivariable Cox regression analyses of the determinants of duration of watchful waiting, postdiagnosis survival and postchemotherapy survival.**

**Characteristics**	**Duration of watchful waiting**		**Postdiagnosis survival**		**Postchemotherapy survival**	

**Crude HR (95% CI)**	**Adjusted HR (95% CI)**	**Crude HR (95% CI)**	**Adjusted HR (95% CI)**	**Crude HR (95% CI)**	**Adjusted HR (95% CI)**	
Binet stage						

A	1.00	1.00	1.00	1.00	1.00	1.00

B	**3.66 (1.77–7.56)**	**4.01 (1.89–8.51)**	2.06 (0.49–8.71)	N/A	0.85 (0.20–3.56)	N/A

C	**6.46 (3.02–13.82)**	**5.28 (2.26–12.34)**	3.19 (0.56–18.06)	N/A	1.37 (0.25–7.65)	N/A

Sex						

Female	1.00	1.00	1.00	1.00	1.00	1.00

Male	1.64 (0.90–3.00)	N/A	1.31 (0.40–4.30)	N/A	0.59 (0.16–2.21)	N/A

Age (per year increase)	1.02 (0.99–1.04)	N/A	**1.12 (1.04–1.21)**	**1.26 (1.09–1.46)**	**1.13 (1.04–1.22)**	**1.13 (1.04–1.22)**

Lymphocytes (per 1000/mm^3^ increase)	**1.008 (1.004–1.012)**	**1.005 (1.001–1.009)**	**1.009 (1.002–1.017)**	**1.014 (1.003–1.024)**	1.005 (0.996–1.013)	N/A

Platelets (per 100,000/mm^3^ increase)	**0.48 (0.32–0.73)**	N/A	0.53 (0.23–1.20)	N/A	0.86 (0.34–2.16)	N/A

PLR (per unit increase)	**0.97 (0.94–0.99)**	N/A	0.95 (0.89–1.02)	N/A	0.98 (0.92–1.05)	N/A

For multivariable models (adjusted HR), variables not included in the stepwise model had N/A in the table grid.

Hazard ratios with an associated p-value < 0.05 were highlighted in bold.

95% CI: 95% Confidence interval; HR: Hazard ratio; N/A: Not applicable; PLR: Platelet-to-lymphocyte ratio.

Patients with increasing age were found to have significantly decreased postdiagnosis survival (HR = 1.12 [1.04–1.21]; p = 0.002) and postchemotherapy survival (HR = 1.13 [1.04–1.22]; p = 0.003). For each increase of the lymphocyte count by 1000/mm^3^, patients had significantly decreased postdiagnosis survival (HR = 1.009 [1.000–1.017]; p = 0.009).

In the multivariable analysis, the duration of watchful waiting was found to be solely determined by Binet staging (B vs A: HRa = 4.01 [1.89–8.51], p < 0.001; C vs A: HRa = 5.28 [2.26–12.34]; p < 0.001) and lymphocyte count (HRa = 1.005 [1.001–1.009]; p < 0.001). The postdiagnosis survival was found to be determined by age (HRa = 1.26 [1.09–1.46]; p = 0.002) and lymphocyte count (HRa = 1.014 [1.003–1.024]; p = 0.010). The postchemotherapy survival was found to be solely determined by age (HRa = 1.13 [1.04–1.22]; p = 0.003).

## Discussion

The CLL is a heterogeneous disease with a variable clinical course that has been historically predicted according to the Rai and Binet staging systems [7,8]. However, they do not take into account other biological characteristics of CLL cells that may influence the disease course. To date, the advent of major molecular breakthroughs has led to the adoption of complex and expensive biomarkers such as cytogenetic abnormalities (trisomy 12, 11q deletions and 17p deletions), β2 microglobulin, thymidine kinase, CD38 and ZAP-70 expression, and IGHV mutation status, and mutations in genes such as *NOTCH1*, *MYD88*, *SF3B1* and *ATM* are also predictors of prognosis [9]. These novel molecular advances serve the ancient hallmarks of cancer and under-recognize the current trend in the concepts of carcinogenesis [10]. Unfortunately, these new biomarkers were not performed in this retrospective series and could not be analyzed with regards to PLR. Another limitation to this analysis would be auto-immune induced thrombocytopenia that may have affected the platelet count. However, to the best of our knowledge, patients with auto-immune hematologic manifestations were not included in this analysis. Only one patient presented extreme thrombocytopenia and his work up did not reveal an immune phenomenon.

The conceptual progresses of the last decade have recognized inflammation as a major hallmark in carcinogenesis development and progression [11]. The evidence connecting inflammation and cancer is now clearly established with the description of inflammatory cytokines that affect carcinogenesis, dedifferentiation and primary tumor growth [12]. As such, several inflammatory biomarkers have been investigated for cancer prognostication [13]. Some of these factors were converted into ratios such as the Glasgow Prognostic Score, the neutrophil to lymphocyte ratio, and the PLR [5,14]. In the particular case of PLR, its prognostic value has been confirmed in solid tumors where it was found to be correlated to OS and to cancer aggressiveness [15]. Moreover, among hematologic malignancies, PLR has only been evaluated in multiple myeloma [16]. Jung *et al*. have reported that the inverse PLR had a predictive value for progression-free survival and OS in the uni- and multi-variable analyses of patients with multiple myeloma treated with novel agent-containing regimens [16].

Molica *et al*. have reported that the absolute count of lymphocytes doubling time was an independent prognostic factor for clinical outcomes in patients with newly diagnosed CLL. However, the prognostic role of platelet count is not well known in CLL although a low platelet count may be an indication for treatment initiation [6]. Although debatable, thrombocytopenia causes a physiologic compensatory secretion of thrombopoietin that seems to correlate with other prognostic biomarkers including IGHV mutation status, ZAP 70 and CD38 [17,18]. Consequently, we hypothesized that the PLR is an easily adapted clinical tool that could segregate the poor prognostic patients earlier so that earlier interventions can be tested before tumor burden makes such interventions unlikely to succeed.

## Conclusion & perspective

The biological rationale in calculating PLR stems from the increase in the lymphocyte count and reduction in the platelet count often encountered in the advances stages of CLL [6]. Therefore, we hypothesized that the ratio using both the platelet and lymphocyte counts may have a prognostic role in patients with CLL. The PLR correlated to the duration of watchful waiting in the univariable analysis only. In this retrospective study, some patients were not assessed for cytogenetic analysis or fluorescence *in situ* hybridization. Therefore, cytogenetic risk factors were not included in the uni- and multi-variable for survival outcome. These data need to be validated in a large number of patients with samples and survival data collected in a uniform fashion along with molecular analysis. We believe that a prospective study taking into consideration the methodological, physiological and pathological confounding factors should better assess the role of PLR in CLL. The PLR biomarker relates to the pathogenesis and clinical implications of CLL, which may be a surrogate for treatment indication especially in cases of cytogenetic and molecular analysis unavailability.

**Executive summary**

**Aim**

- Platelet levels tend to be decreased and lymphocyte levels are increased in patients with chronic lymphocytic leukemia (CLL).
- The rationale for platelet-to-lymphocyte ratio (PLR) as a biomarker for CLL prognosis is that these alterations in platelet and lymphocyte counts could predict the survival of patients with CLL.

**Methods**

- Demographic and clinical data of CLL patients diagnosed at our institution between 1989 and 2013 were collected.
- The duration of watchful waiting, postdiagnosis survival and postchemotherapy survival for evaluated for all patients.
- Univariable and multivariable Cox regression models were used to evaluate the role of PLR as a determinant of duration of watchful waiting, postdiagnosis survival and postchemotherapy survival.

**Results**

- The data of 100 patients with CLL were reviewed for this study.
- The PLR correlated only to watchful waiting in the univariable analysis (HR = 0.48 [0.32–0.73]; p = 0.018).
- In the multivariable analysis, the duration of watchful waiting was determined by Binet staging and lymphocyte count (p < 0.001).
- The postdiagnosis survival was determined by age (p = 0.002) and lymphocyte count (p = 0.010).
- The postchemotherapy survival was found to be solely determined by age (HRa = 1.13 [1.04–1.22]; p = 0.003).
- However, both postdiagnosis survival and postchemotherapy survival were not found to be related to PLR.

**Conclusion**

- The PLR was only found to be related to the duration of watchful waiting.
- In this study, PLR was not found to be significantly related to the survival of patients with CLL.
- The PLR does not seem to be viable biomarker for survival in patients with CLL.
